# Olfactory response of *Mahanarva spectabilis* (Hemiptera: Cercopidae) to volatile organic compounds from forage grasses

**DOI:** 10.1038/s41598-019-46693-9

**Published:** 2019-07-16

**Authors:** Sandra E. B. Silva, Alexander M. Auad, Jair C. Moraes, Roberta Alvarenga, Marcy G. Fonseca, Francisco A. Marques, Nayana C. S. Santos, Noemi Nagata

**Affiliations:** 1 Universidade Federal de Lavras, Caixa postal 3037, Cep 37200000, Lavras, MG Brazil; 2 Embrapa Gado de Leite, Av. Eugênio do Nascimento, 610, CEP 36038-330, Juiz de Fora, MG Brazil; 3 Universidade Federal do Paraná, Rua XV de Novembro, 1299, CEP 80060-000, Curitiba, PR Brazil

**Keywords:** Ecology, Chemical biology

## Abstract

Several herbivorous insects utilize plant chemical cues to identify hosts for feeding. The role of smell in host plant detection by *Mahanarva spectabilis* (Distant) remains largely unknown. In this study, assays were applied to assess *M*. *spectabilis* olfactory responses to forage grasses (*Pennisetum purpureum* cvs. Roxo Botucatu and Pioneiro; *Panicum maximum* cvs. Makueni and Tanzânia; *Hyparrhenia rufa* cv. Jaraguá; *Melinis minutiflora*; *Cynodon dactylon* cv. Tifton; *Brachiaria brizantha* cv. Marandú; and *Brachiaria decumbens* cv. Basilisk). Bioassays were performed using a Y-olfactometer to evaluate the behavior of adult *M*. *spectabilis* to forage damaged and undamaged by insects. *M*. *spectabilis* preferred volatiles of undamaged Basilisk and Pioneiro. Repellent behavior by *M*. *spectabilis* to cospecifics was recorded for plant volatiles from damaged Marandú. The mixture of volatiles from undamaged forage grasses differed from that of forage grasses damaged by insects. Forage grasses showed a greater diversity of compounds after damage, including menthone, eucalyptol and camphor, which are compounds likely to cause loss of attractiveness or repellence. Our results demonstrate that *M*. *spectabilis* employs plant chemical cues in its choice of hosts. This fact may contribute to strategies of integrated management against this pest.

## Introduction

Spittlebugs (Hemiptera: Cercopidae), which are pests found on forage grasses of tropical America, impair plant growth and lead to low production and poor quality^1^. When sucking plant sap, adult insects inject toxins that interfere with photosynthetic activity, resulting in a yellowish color and dwindled leaves and possibly even causing death^2^.

Worldwide loss due to spittlebugs may reach between US$ 840 million and US$ 2.1 billion dollars annually^3^. In the case of Brazil, *Mahanarva spectabilis* (Distant) is considered a constraint to the production of forage grasses^4^; it is responsible for severe attacks and impairs beef and milk production chains because most Brazilian cattle feed solely on pasture^5^.

However, chemical insecticide-based control is not recommended for spittlebugs, as it is neither ecologically nor economically feasible due to the required treatment of extensive areas and its high costs, respectively^6^. Plant resistance may therefore be a good alternative strategy because it decreases pest populations without interfering with the ecosystem and without additional production costs.

In endeavors to identify spittlebug-resistant forage grasses, a recent study on the performance and feeding behavior of *M*. *spectabilis* Silva *et al*.^7^ revealed antixenosis or nonpreference resistance mechanisms of *Melinis minutiflora* and *Panicum maximum* cv. Makueni and Tanzânia against *M*. *spectabilis*. The authors suggested that the insect nonpreference for these plants and its preference for others, such as *Brachiaria decumbens* cultivars Jaraguá, Roxo de Botucactu and Pioneiro, may be related to volatile compounds released by the plants.

Each plant species releases a mixture of specific volatile organic compounds that play crucial roles in ecological interactions with other organisms^8^. These compounds are components of the plant defense system against herbivore attack^9^, although they may also be involved in guiding herbivore preference for food and oviposition^10^ as well as in insect repellent strategies^11,12^.

Several studies have focused on chemical communication between plants and herbivore insects. Indeed, analysis of this type of communication is greatly relevant because the compounds involved have potential for integrated pest management due to their interactivity mediation^13^. Therefore, the use of semiochemicals may generate alternative or auxiliary measures for conventional control methods and population monitoring^14^. For example, the push-pull system successfully employs plant volatile compounds in pest management^15^.

There is little knowledge to date about the olfactory role of forage grass volatile compounds on the behavior of spittlebugs with regard to their choice of host plants, yet elucidation of the olfactory basis of behavior in host plants may significantly contribute to a better understanding of olfactory reception and perception. This knowledge may be applied to plant-associated volatile compounds for integrated management of spittlebugs. The present study evaluates the olfactory responses of *M*. *spectabilis* to different forage plants and identifies the response-interfering chemical compounds released by these forage plants.

## Results

### Olfactometer bioassays

**Figure 1:**
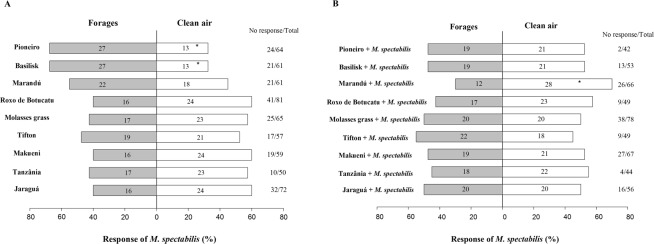
Behavioral response of *M*. *spectabilis* adults to undamaged (**A**) or damaged (**B**) plant volatiles or clean air using a Y-olfactometer. (*) denotes a significant difference (p < 0.05). The numbers inside the bars are the total numbers of spittlebugs that responded to each treatment.

*Mahanarva spectabilis* was attracted by cvs. Basilisk (χ² = 4.9; GL = 1; P = 0.027) and Pioneiro (χ² = 4.9; GL = 1; P = 0.027) when undamaged. The odorants of undamaged forage cvs. Jaraguá, Tanzânia, Makueni, Tifton, Molasses, Roxo de Botucatu and Marandú were not significantly attractive or repellent to spittlebugs (Fig. 1A).

No forage species attacked by the pest released odorants that would significantly attract *M*. *spectabilis*. Repellent odors only produced responses by *M*. *spectabilis* in response to damaged Marandú (χ² = 6.4; GL = 1; P = 0.011) (Fig. 1B).

### Chemical analysis

**Figure 2:**
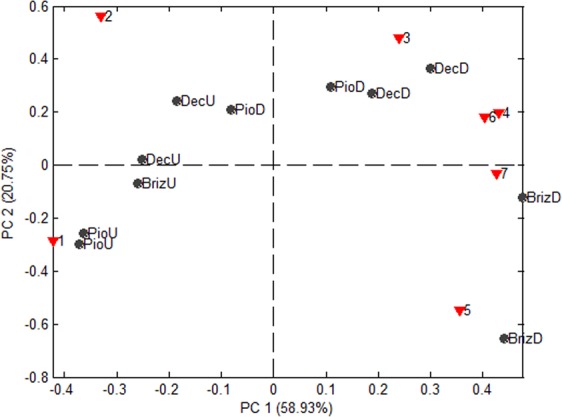
Principal component analysis of the profile of volatiles from Pioneiro (Pio), Basilisk (Dec) and Marandú (Briz), undamaged (U) or damaged (D) by *M*. *spectabilis*. The score (●) and loading (▼) of PCA were based on the percentages of compounds in all volatile mixtures. The first and second PCs accounted for 58.93% and 20.75% of the total variation, respectively. Each point in the score represents a replication. Number on the loading refers to compounds: 1-limonene, 2-β-pinene, 3-α-pinene, 4-menthone, 5-camphor, 6-eucalyptol, and 7-*o*-cymene.

Chemical analysis revealed that volatiles from cultivars Pioneiro, Basilisk and Marandú, damaged and undamaged by *M*. *spectabilis*, differed (Table 1). PCA and the distribution of scores corroborated this fact (Fig. 2). Damaged and undamaged cultivars were separated by the first main component (PC1), with 58.93% of total data variance. Although the second component (PC2) provided a relevant 20.72% variation, totaling approximately 80% of the variance, the sample distribution between PC1 and PC2 provided indices for the discrimination of cultivars.

**Table 1:** Mean (±SE) volatile compounds (ng) identified from extracts collected from foragers Pioneiro, Marandú and Basilisk, undamaged and damaged by *M*. *spectabilis*

Compounds	Pioneiro undamaged	Basilisk undamaged	Marandú undamaged	Pioneiro damaged	Basilisk damaged	Marandú damaged
1- Limonene	54.03 ± 0.7	55.33 ± 1.9	50.25 ± 0.0	52.55 ± 3.4	53.94 ± 0.1	38.78 ± 5.7
2- β-Pinene	45.97 ± 0.7	41.85 ± 2.5	46.82 ± 0.0	41.16 ± 1.0	37.07 ± 1.1	37.98 ± 2.0
3- α- Pinene	ND	2.81 ± 0.6	2.93 ± 0.0	3.03 ± 0.8	2.74 ± 0.0	2.46 ± 0.4
4- Menthone	ND	ND	ND	1.69 ± 1.2	1.92 ± 0.7	1.54 ± 0.4
5- Camphor	ND	ND	ND	ND	1.04 ± 0.2	15.49 ± 5.2
6- Eucalyptol	ND	ND	ND	2.3 ± 0.3	1.53 ± 0.2	2.15 ± 0.7
7- *o*-Cymene	ND	ND	ND	ND	1.76 ± 0.0	1.59 ± 0.0

^ND^not detected.

Overall analysis of score graphs and PC1 vs. PC2 loadings (Fig. 2) revealed that the undamaged forage volatile mixture could be characterized by the release of limonene and β-pinene at relatively high concentrations. However, these compounds were also found in damaged forage. Consequently, the absence of menthone, camphor, eucalyptol and *o*-cimene is the main identifier of undamaged plants.

The compounds menthone and eucalyptol were found in extracts from damaged Marandú, repelling insects in olfactometry tests, and Basilisk and Pioneiro, which lost their attractiveness when compared to undamaged plants. Marandú and Basilisk released camphor and *o*-cimene. Although cultivars Marandú and Basilisk damaged by the insect pest exhibited the same volatile constitution, the former released a greater amount of camphor (Table 1). This fact may be observed by the positioning of the cultivar (positive side of PC1 and PC2) with the greatest influence by variable 5 (Camphor) (Fig. 2).

## Discussion

Several insects employ plant volatile organic compounds as olfactory cues in the process of finding a host plant and are able to discriminate between host and nonhost plants^16^. Here, the role of volatiles from forage plants in the behavior of adult *M*. *spectabilis* was investigated, and the responses of this insect were often based on the forage species/cultivars and plant induction level.

Damaged and undamaged plants of Roxo de Botucatu, Molasses grass, Makueni, Tanzânia, Jaraguá and Tifton failed to change the response behavior of *M*. *spectabilis*, as measured by an olfactometer. The nonresponse to these plants may be associated with the low specificity of *M*. *spectabilis* to volatiles released. On the other hand, insect responses to Basilisk, Pioneiro (attraction by undamaged plant) and Marandú (repellence by damaged plant) reveal that olfactory cues may be involved in host plant selection by *M*. *spectabilis*.

*Mahanarva spectabilis* adults responded positively to volatile undamaged Pioneiro and Basilisk plants, confirming the feed preference of *M*. *spectabilis* for these cultivars, as suggested by Silva *et al*.^7^, who proved the insect attraction to Basilisk in a greenhouse and to Pioneiro in both a greenhouse and in the field. Insect attraction to released volatiles suggests that they receive information from the plants to detect adequate food sources for their survival. This fact is corroborated by the susceptibility of plants to nymphs and adults of this species, as demonstrated by Silva *et al*.^7^.

In contrast, repellent behavior may occur if the host’s odor reveals a poor-quality host^15^, as is the case of Marandú, which is resistant to *M*. *spectabilis*^17,18^, and in the current study was the only plant that repelled the insect pest when damaged. Previous greenhouse studies have also revealed that after one hour of infestation by *M*. *spectabilis*, Marandú was ignored for feeding and was hardly considered attractive in the field^7^. Other studies have also shown low attraction of herbivore insects to plants infested by cospecifics. For instance, Da Costa *et al*.^19^ detected that *Capsicum* spp. cultivar SPHGB repelled the aphid *A*. *gossypii* after infestation by cospecifics.

*Mahanarva spectabilis* failed to respond to volatiles released by damaged plants of Basilisk and Pioneiro. Hence, this insect may discriminate between infested and noninfested plants. *M*. *spectabilis* also discriminated between volatiles of damaged and undamaged Marandú. When herbivores feed on a plant, volatile organic compounds are released from the damage site due to tissue damage^8^. Studies have shown that biochemical and physiological changes in plants after herbivore insect feeding can modify the constitution of the volatiles released^20,21^. PCA in the present study revealed a sharp difference between the volatiles released before and after herbivory (Table 1; Fig. 2), i.e., menthone, eucalyptol, camphor and o-cimene released only by damaged plants. These compounds are monoterpenes that, together with other terpenoids, are one of the major herbivore-induced plant volatile (HIPV) groups^22^, and their release after pest infestation has been studied. For instance, Fernandes *et al*.^23^ detected camphor emission by kale after herbivory by *Pieris brassicae* L. Another study revealed the release of eucalyptol by the roots of Arabidopsis after herbivory by *Diuraphis noxia* (Mordvilko)^24^.

Although damaged Pioneiro and Basilisk were not attractive to *M*. *spectabilis*, the plants’ volatile compounds differ: Basilisk contained o-cimene and camphor, but Pioneiro did not. Menthone and eucalyptol were the common compounds released by these two forage cultivars after herbivory. We are of the opinion that these compounds affect host selection behavior and, by making plants less attractive to insects, may be capable of reducing colonization by additional herbivores. In fact, studies have reported that these compounds may have insecticide or deterrent activities^25,26^.

The chemical analysis showed that although the compounds released by Basilisk and Marandú were qualitatively the same, there was a greater production of camphor by Marandú. Camphor has been shown to be one of the compounds that repel other insects, such as the moth *Ectropis obliqua* Prout^11^. Thus, there is evidence that this compound is a cause of repellence to *M*. *spectabilis*. Repellent plants contain a key component in push-pull strategies based on plants for the management of pest populations^11^. For instance, in Africa, molasses grass and *Desmodium uncinatum* (Jacq), which release repellent volatiles, are interspersed between maize crops to reduce pest populations^27,28^.

It was verified a large number of nonresponding insects, despite their ability to respond to olfactory stimuli. This situation may be explained by the fact that the period of *M*. *spectabilis* occurrence and test performance coincided with the rainy season in Brazil, with a fall in barometric pressure. It has already been observed that decreased barometric pressure alters insect behavior^29^.

The current analysis is the first to show that olfactory cues may have a role in interspecies communication between spittlebug and forage plants. It was demonstrated that the choice of a host plant may be attributed to specific odors of plants (such as Basilisk and Pioneiro). Moreover, the repellence of damaged plants (Marandú) suggests the potential employment of these plants in management programs for *M*. *spectabilis*; this is especially true for the compound camphor, which apparently repels spittlebugs, and other volatiles that render forage grasses less attractive, such as menthone and eucalyptol. Identification of these volatiles was the first step in understanding the ecological roles of these compounds in chemical communication between forage plants and spittlebugs.

## Materials and Methods

### Maintaining insects and plants

Adult *M*. *spectabilis* specimens were collected weekly from the experimental field of Embrapa Gado de Leite in the municipality of Coronel Pacheco MG Brazil. Adults were fed elephant grass (*Pennisetum purpureum* Schum cv. Napier) in acrylic cages (30 × 30 × 60 cm) under controlled conditions (25 ± 2 °C, 70–80% relative humidity). The following forage grasses were used in the experiments: *P*. *purpureum* Schum cvs. Roxo de Botucatu and Pioneiro; *Panicum maximum* Jacq. cvs. Makueni and Tanzânia; *Hyparrhenia rufa* (Nees) Stapf (Jaraguá); *M*. *minutiflora* Beauv. (molasses grass); *Cynodon dactylon* (L.) Pers cv. Tifton; *Brachiaria brizantha* (Hochst ex A. Rich Stapf) cv. Marandú; and *B*. *decumbens* Stapf cv. Basilisk. Plant seedlings from greenhouses were singly planted in 300-mL plastic cups in soil with a clayey texture (59% clay, 5% silt, 36% sand).

The plants were grown in a greenhouse until 30–35 days old and then used for olfactometry bioassays and collection of volatile compounds. Continuous planting occurred at regular intervals.

### Olfactometer bioassays

Olfactometry bioassays were performed at Entomology Laboratory at Embrapa Gado de Leite, Juiz de Fora, MG. A glass Y-type olfactometer (3.5 cm diameter, main arm measuring 30 cm; side arms measuring 23 cm each; angle 120° between arms) with a continuous air flow at 1.0 L/min was used, as described for Saraiva *et al*.^30^. Pumped air was humidified with distilled water, filtered with activated coal and calibrated by a flow meter. Each arm of the olfactometer was linked by silicone tubes to two glass chambers (42 cm high x 16 cm wide): one contained vegetal material, and the other was a control (clean air).

To reduce possible effects of volatile substances emitted from the soil in which the plants grew, the plastic cups containing plants were wrapped in aluminum sheets from the cup bottom to the stem of the plant. All plant varieties were tested against clean air. The plants were undamaged or damaged by *M*. *spectabilis* as follows: (1) Pioneiro undamaged *vs*. clean air; (2) Roxo de Botucatú undamaged *vs*. clean air; (3) Basilisk undamaged *vs*. clean air; (4) Marandú undamaged *vs*. clean air; (5) elephant grass undamaged *vs*. clean air; (6) Jaraguá undamaged *vs*. clean air; (7) Tanzânia grass undamaged *vs*. clean air; (8) Makueni undamaged *vs*. clean air; and (9) Tifton undamaged *vs*. clean air.

Olfactory responses of the forage species damaged by *M*. *spectabilis* adults *vs*. clean air were also assessed. For damage, the plants were exposed to four couples of *M*. *spectabilis* maintained in nylon cages (35 × 60 cm) for 24 hours. Thirty minutes prior to the bioassay, the insects were removed from plants to avoid interference of herbivore response by semiochemicals emitted by the insects.

Prior to olfactometry bioassays, adult insects were removed from the nylon cages and kept in voile fabric cages for one hour without food. The insects were subjected to individual tests by placing them at the olfactometer base. A response was considered to have occurred when the insect rushed against the air flow and reached the end of one of the Y arms within 10 minutes. Insects that failed to respond during this time interval were tagged as nonresponsive and were not included in the analysis.

Responses by at least 40 insects were evaluated for each forage grass. Each specimen was tested once to avoid pseudorepetition. Plants within the same treatment were exchanged with different plants in 10-insect intervals to replenish the odorant source. After five insects were tested, the olfactometer was washed with ethyl alcohol 96° GL and soaked in a buffer at 100 °C for ten minutes. The olfactometer was also rotated at 180° to avoid positional bias. After ten insects were tested, the olfactometer was washed with detergent, distilled water and alcohol and placed in a buffer at 100 °C for 20 minutes. All tests were performed between 10 hours and 16 hours during the insect occurrence period (between October and April) for 2015 and 2016. The mean temperature during the testing period was 26 ± 2 °C, and the relative humidity was 60 ± 10%.

### Air entrainment of plant volatiles

An aeration technique was employed for volatile collection from forage. Plants (cvs. Basilisk, Pioneiro and Marandú), which altered the response of *M*. *spectabilis* in the olfactometer tests, were selected for extraction. To examine possible changes in the constitution of volatile compounds, volatiles were collected from these plants damaged and undamaged by *M*. *spectabilis*.

The 300-mL cups containing plants were wrapped in aluminum paper as described above and placed individually in a glass chamber (42 cm high × 16 cm wide) adapted for aeration, in which a continuous 1.0 L/m flow of humidified, activated, coal-filtered air calibrated by a fluxometer passed through the chamber carrying volatiles released by the plant. The volatiles remained in the glass column (11 cm long × 1 cm diameter) by using 0.5 g of adsorbent polymer (Haye Sep® D 80/100 Supelco, Belfonte PA), according to Zarbin^31^.

Volatile compounds released by the plants were collected over 24 hours, and each treatment was repeated ten times. The compounds were desorbed from the adsorbent using 4 mL of distilled *n*-hexane (J. T Backer® 95% hexane, Sovereign, Taboão da Serra SP Brazil) into borosilicate glass vial. The samples were stored in vials at −25 °C until used in the chemical analyses.

### Chemical analysis

#### Gas chromatography mass spectrometry (CG/MS)

Analysis was performed using a Shimadzu CGMS-QP2010 Plus system equipped with a quadrupole mass detector with a Rtx-5MS (Crossbond 5% diphenyl/95% dimethylpolysiloxane) low-bleeding column (30 m × 0.25 mm × 0.25 μm), with helium as the carrier gas at a flow rate of 1.02 mL/min. 1 µL of the sample was injected splitless at an initial oven temperature of 60 °C. The injector and detector temperatures were adjusted to 250 °C. The programmed oven temperature was 60–250 °C at 3 °C/min.; EIMS: electron energy, 70 eV; ion source temperature and connection parts at 180 °C.

#### Peak identification

Individual components were identified by comparing retention indices (RIs) and mass spectra with those of authentic compounds given in Adams Libraries of mass spectral data^32^ and by a computer database using Wiley 275, NIST 21, NIST 107^33^.

### Statistical analysis

The choices by each spittlebug were analyzed using the χ^2^ test within the R Core Team^34^. Insects that did not choose any of the arms were excluded from the statistical analysis.

Quantity data of volatile organic compounds (relative %) extracted from cultivars Pioneiro, Basilisk and Marandú, undamaged or damaged by *M*. *spectabilis* (Table 1), were processed by Principal Components Analysis (PCA) using PLS-Toolbox 3.0, operating in MATLAB 7.0.1. Data were autoscaled to compensate for differences between concentrations of each compound and to avoid camouflage of minor volatile compounds.

## Data Availability

The datasets generated and/or evaluated during the current study are available from the corresponding author on request.
